# Editorial: Cellular and network mechanisms underlying behavioral functions of the prefrontal cortex and reward circuitry pertaining to psychopathology

**DOI:** 10.3389/fncel.2022.995495

**Published:** 2022-08-02

**Authors:** Jonna M. Leyrer-Jackson, Mark P. Thomas, M. Foster Olive

**Affiliations:** ^1^Department of Medical Education, School of Medicine, Creighton University, Phoenix, AZ, United States; ^2^School of Biological Sciences, University of Northern Colorado, Greeley, CO, United States; ^3^Department of Psychology, Arizona State University, Tempe, AZ, United States

**Keywords:** prefrontal cortex, psychopathology, addiction, cell network, reward

It is well known that psychopathologies such as substance abuse, schizophrenia, disorders of attention, and anxiety disorders, among others, are associated with abnormal neurophysiology across multiple brain regions including the prefrontal cortex (PFC). Abnormal reward processing is a prominent feature of various psychopathologies, and normal function is reliant on optimal levels of dopamine and excitability within the prefrontal cortex. While many studies have explored the relationships between pathological cognition and behavior and the underlying neurophysiological properties of cellular networks across brain regions, it remains of high importance to uncover additional pathways and mechanisms which may be targeted for therapeutic treatments and alleviation of associated symptomology.

In this Research Topic entitled “Cellular and Network Mechanisms Underlying Behavioral Functions of the Prefrontal Cortex and Reward Circuitry Pertaining to Psychopathology” we, together with leaders in the field, have brought together the most recent findings and insights into the role of neuronal cell populations and pathways that give rise to phenotypical behaviors associated with substance abuse, contextual processing, and dopamine-mediated behaviors. Our collection includes 7 research articles that address several different reward pathways and their contributions to behavior, as well as the role of different receptor subtypes located within the central and peripheral nervous systems that modulate behavioral phenotypes. Four of the articles focus on substance abuse and specific neuronal cell types and pathways that may be altered and ultimately promote relapse. One study highlights the effects of heroin, methamphetamine, cocaine and the synthetic cathinone MDPV on layer V pyramidal cells within the PFC, demonstrating that each hinders the excitability of contralaterally projecting subtypes (Leyrer-Jackson et al.). Gonzalez et al. report findings that perineuronal nets (PNN), which surround parvalbumin positive GABAergic neurons within the PFC, regulate cocaine-associated contextual memories, and removal of PNNs in this region alters GABAergic neuronal activity and inhibits reinstatement of cocaine induced place preference. Further, Siemsen et al. found that prelimbic PFC projections targeting the nucleus accumbens are required for cue-induced relapse to cocaine seeking. Taken together, these studies have shed light on additional prefrontal mechanisms that may promote cocaine seeking and substance abuse. Another contribution reports that chronic intermittent ethanol and withdrawal alters the excitability of neurons in the basolateral amygdala targeting the bed nucleus of the stria terminalis (BNST) and the nucleus accumbens (NAc) (Price and McCool). Their findings are among the first to demonstrate ethanol's effects on glutamatergic projections between these regions that may facilitate alcohol intake and anxiety-like behaviors, respectively.

Other contributions to this Research Topic focus on the effects of catecholamines in mediating contextual information processing. Specifically, enhancing noradrenaline release and the activation of β-adrenergic receptors within the dorsal CA1 was found to enhance contextual associative learning (Tsetsenis et al.), and thus may be important in treating cognitive dysfunction observed across different disease states. Further, peripheral D2 receptor inhibition was found to reduce intravenous dopamine conditioned place preference and dopamine levels within the NAc (Obray et al.), an area important in regulating reward and reward-associated behaviors, suggesting that peripheral dopamine receptors also play a role in mediating dopamine-regulated behaviors. In addition, a computational model exploring existing physiological experiments as they relate to major depressive disorder and the role of NAc-PFC-ventral tegmental area regional crosstalk was elegantly designed to be applicable to other neuropsychiatric disorders and to enhance our current understanding of this important neural circuitry (Li et al.).

The articles presented in this Research Topic provide interesting insights into multiple cellular and network mechanisms that contribute to various psychopathologies. Each study highlights important findings that contribute to our current understanding and reveal novel insights into neurophysiological mechanisms ranging from receptors to cell types and pathways, all of which may prove important for future therapeutic development. Together, these studies also underscore the need for additional exploration of neurophysiological pathways, especially related to reward processing, that contribute to complex behaviors observed across various disease states.

## Author contributions

All authors listed have made a substantial, direct, and intellectual contribution to the work and approved it for publication.

## Funding

This work was supported by Public Health Service grants F32AA027962 to JL-J, as well as DA043172 and AA025590 to MO.

## Conflict of interest

The authors declare that the research was conducted in the absence of any commercial or financial relationships that could be construed as a potential conflict of interest.

## Publisher's note

All claims expressed in this article are solely those of the authors and do not necessarily represent those of their affiliated organizations, or those of the publisher, the editors and the reviewers. Any product that may be evaluated in this article, or claim that may be made by its manufacturer, is not guaranteed or endorsed by the publisher.

